# The Regulate your Sitting Time (RESIT) intervention for reducing sitting time in individuals with type 2 diabetes: findings from a randomised-controlled feasibility trial

**DOI:** 10.1186/s13098-024-01336-6

**Published:** 2024-04-24

**Authors:** Marsha L. Brierley, Angel M. Chater, Charlotte L. Edwardson, Ellen M. Castle, Emily R. Hunt, Stuart JH. Biddle, Rupa Sisodia, Daniel P. Bailey

**Affiliations:** 1 Centre for Physical Activity in Health and Disease, College of Health, Medicine and Life Sciences, Brunel University London, Kingston Lane, UB8 3PH Uxbridge, UK; 2 Division of Sport, Health and Exercise Sciences, Department of Life Sciences, Brunel University London, Kingston Lane, UB8 3PH Uxbridge, UK; 3 Institute for Sport and Physical Activity Research, Centre for Health, Wellbeing and Behaviour Change, University of Bedfordshire, Polhill Avenue, MK41 9EA Bedford, UK; 4 Centre for Behaviour Change, University College London, 1-19 Torrington Place, WC1E 7HB London, UK; 5 Leicester Lifestyle and Health Research Group, Diabetes Research Centre, University of Leicester, Leicester General Hospital, LE5 4PW Leicester, UK; 6 NIHR Leicester Biomedical Research Centre, Leicester General Hospital, LE5 4PW Leicester, UK; 7 Physiotherapy Division, Department of Health Sciences, College of Health, Medicine and Life Sciences, Brunel University London, Kingston Lane, UB8 4PH Uxbridge, UK; 8 Curtin School of Allied Health, School of Health Sciences, Curtin University, Western Australia, 6845 Bentley, Australia; 9 Centre for Health Research, University of Southern Queensland, Springfield Central, 4300 Springfield, QLD Australia; 10 Faculty of Sport & Health Sciences, University of Jyväskylä, FI-40014 Jyväskylä, Finland

**Keywords:** Sedentary behaviour, Prolonged sitting, Physical activity, Behaviour change, Diabetes, ActivPAL, Mixed methods

## Abstract

**Background:**

Reducing and breaking up sitting is recommended for optimal management of Type 2 diabetes mellitus (T2DM). Yet, there is limited evidence of interventions targeting these outcomes in individuals with this condition. The primary aim of this study was to assess the feasibility and acceptability of delivering and evaluating a tailored online intervention to reduce and break up sitting in adults with T2DM.

**Methods:**

A mixed-methods two-arm randomised controlled feasibility trial was conducted in ambulatory adults with T2DM who were randomised 1:1 to the REgulate your SItting Time (RESIT) intervention or usual care control group. The intervention included online education, self-monitoring and prompt tools (wearable devices, smartphone apps, computer apps) and health coaching. Feasibility outcomes were recruitment, attrition, data completion rates and intervention acceptability. Measurements of device-assessed sitting (intended primary outcome for definitive trial), standing and stepping, and physical function, psychosocial health and wellbeing were taken at baseline, 3 months and 6 months. Individual semi-structured interviews were conducted at six-months (post intervention) to explore acceptability, feasibility and experiences of the trial and intervention using the Framework Method.

**Results:**

Seventy participants aged 55 ± 11 years were recruited. Recruitment rate (proportion of eligible participants enrolled into the study) was 67% and participant retention rate at 6 months was 93% (*n* = 5 withdrawals). Data completion rates for daily sitting were 100% at baseline and ranged from 83 to 91% at 3 months and 6 months. Descriptive analysis demonstrated potential for the intervention to reduce device-measured sitting, which was 30.9 ± 87.2 and 22.2 ± 82.5 min/day lower in the intervention group at 3 and 6 months, respectively, compared with baseline. In the control group, sitting was 4.4 ± 99.5 and 23.7 ± 85.2 min/day lower at 3 and 6 months, respectively. Qualitative analysis identified three themes: reasons for participating in the trial, acceptability of study procedures, and the delivery and experience of taking part in the RESIT intervention. Overall, the measurement visits and intervention were acceptable to participants.

**Conclusions:**

This study demonstrated the feasibility and acceptability of the RESIT intervention and evaluation methods, supporting a future definitive trial. If RESIT is found to be clinically effective, this could lead to changes in diabetes healthcare with a focus on reducing sitting.

**Trial registration:**

The trial was registered with ISRCTN (number ISRCTN14832389).

**Supplementary Information:**

The online version contains supplementary material available at 10.1186/s13098-024-01336-6.

## Background

Type 2 diabetes mellitus (T2DM) is a major cause of death and accounts for more than 95% of the 422 million people living with diabetes worldwide [1]. People living with T2DM have an increased risk of cardiovascular disease, adverse mental health, disease-related complications such as neuropathy, and are 50% more likely to die prematurely [2, 3]. Personalised care and self-management is recommended for improving glycaemic control and reducing the likelihood of these outcomes in individuals with T2DM [4].

Engaging in ≥ 7 h/day of self-reported sitting was identified as the threshold above which mortality risk starts to increase [5, 6]. When sitting is measured using accelerometry, the risk of mortality increases gradually between 7.5 and 9 h/day and is more pronounced at ≥ 9.5 h/day [7]. Individuals with T2DM accumulate an average of 9.5 to 12.7 h/day of sedentary time (i.e. low energy expenditure while sitting, reclining or lying during waking hours) when measured with accelerometers [8, 9]. Engaging in higher volumes of sedentary behaviour is also adversely associated with cardiometabolic biomarkers and duration of hyperglycaemia in individuals with T2DM, often independent of moderate-to-vigorous and light-intensity physical activity [10–12]. Total daily sitting and prolonged sitting are also related to higher depression scores and reduced quality of life in individuals at high risk of developing T2DM [14]. Furthermore, accumulating sedentary time in prolonged bouts is detrimentally associated with cardiometabolic health, independent of total sedentary time [15]. Breaking up prolonged sitting with regular, short bouts of walking or simple resistance exercises, on the other hand, improves cardiometabolic biomarkers over a single day in ambulatory individuals with T2DM [16, 17]. Reducing daily sedentary time and regularly breaking up prolonged sitting are, therefore, considered important components of 24-hour physical behaviours for T2DM and are recommended for optimal management of this condition [18, 19].

Interventions aimed at reducing and breaking up sitting in office workers and the general adult population have incorporated a wide range of behaviour change techniques (BCTs) [20, 21]. The BCTs of goal setting, problem solving, action planning, self-monitoring, providing information on health consequences, social support, restructuring the physical environment, and providing prompts and cues appear to be acceptable and potentially effective for improving cardiometabolic health, mood and wellbeing in office workers and individuals with T2DM [20, 22]. There is a lack of research evaluating interventions to reduce and break up sitting in people with long-term health conditions, including T2DM [23, 24], meaning their feasibility, acceptability and effectiveness is not well understood. Tailoring interventions to the individual (e.g., through personalised smartphone app features and health coaching with a trained professional) are more likely to lead to improved physical activity and HbA1c outcomes [25, 26]. Enabling individuals to tailor the delivery mode of BCTs could, therefore, enhance engagement and effectiveness of self-management interventions to reduce and break up sitting.

The primary aim of this study was to conduct a randomised controlled feasibility trial to assess the feasibility and acceptability of delivering and evaluating a tailored online intervention to reduce and break up sitting in ambulatory adults living with T2DM. The primary objectives were to evaluate participant recruitment, attrition and data measurement completion rates; acceptability of randomisation to study groups; acceptability of the intervention and data collection procedures; trial safety; and experiences with the intervention. The secondary objective was to evaluate the potential effectiveness of the intervention on sitting, standing, stepping, waist circumference, physical function, mood and wellbeing.

## Methods

### Study design, randomisation, and blinding

This was a mixed-methods randomised controlled feasibility trial, reported following the Consolidation Standards of Reporting Trials statement for pilot and feasibility trials [27]. The study protocol is published [28] and the trial was registered with ISRCTN (ISRCTN14832389). Participants were individually randomised to the REgulate your SItting Time (RESIT) intervention or usual care (control group) after baseline measures. A researcher independent from the study created a computer-generated randomisation list to allocate participants on a ratio of 1:1 (intervention:control) using a fixed block size of four. Researchers and participants were blinded to group allocation until after baseline measures. Blinding thereafter was not possible by virtue of how the intervention was delivered.

### Setting

This study took place from October 2020 to November 2021 in England with community-dwelling individuals with T2DM. Due to the COVID-19 pandemic, lockdown restrictions were in place in the U.K. from October 2020 to February 2021 requiring people to stay at home. A phased easing of lockdown restrictions occurred thereafter with most legal limits on social contact removed in July 2021. All study protocols were carried out remotely due to the COVID-19 pandemic restrictions. Participants were recruited primarily from North West London with some participants being from the Leicester area.

### Eligibility criteria

Participants were eligible if they were aged 18–85 years, diagnosed with T2DM, able to ambulate unassisted, self-reported sitting for ≥ 7 h/day and had access to a smart device or the internet. Due to the COVID-19 pandemic, it was not possible to objectively confirm T2DM status. Instead, each participant’s General Practitioner (GP) was asked to notify the research team if their patient did not have a T2DM diagnosis. Individuals were excluded if they used insulin medication, were unable to communicate in English at a level that would affect their ability to participate fully in the study, were pregnant, or had cognitive or physical conditions that might impede standing and ambulation.

### Sample size

The target sample size was 70, which exceeds recommended sample sizes for pilot and feasibility studies [29]. The number was inflated to provide clearer estimates of feasibility and to explore, in depth, the intervention’s active ingredients and participant experiences. For the nested qualitative analysis, an *a-priori* sample of 13 interviews for each study arm was deemed sufficient to uncover common patterns and themes [30].

### Participant recruitment

Participants were recruited via GP practices (using text messages or mailed study information), local Diabetes UK support groups and social media. General practices were selected and invited to take part by the North West London Clinical Research Network. All potentially eligible patients (i.e. having T2DM diagnosis and being mobile) were approached by each participating GP practice. Interested individuals were screened by email or telephone prior to providing informed consent electronically to take part.

### The RESIT intervention

The RESIT intervention consisted of an online education session, remotely delivered health coaching support, and self-monitoring and prompt tools that were chosen by the participants. It was intended that intervention participants received a core set of BCTs (see Supplementary Material 1) irrespective of which self-selected tools were selected.

#### Online education session

At the start of the intervention all intervention participants were provided with a link to a one-off interactive online education session, adapted from the SMART Work and Life intervention [31]. The session included 19 sections covering the health risks associated with excess sitting, the benefits of reducing and breaking up sitting, reflection on sitting time, the importance of self-monitoring and prompts, goal setting and action planning, and overcoming barriers. Participants could complete this at their own pace but in total it would take ~ 60–90 min to complete.

#### Health coach support

Health coach support was provided to each intervention participant remotely (via video or telephone) approximately 1 to 3 days after gaining access to the online education session (i.e. start of the intervention) and 2, 6 and 12 weeks later. These coaching sessions harnessed the G.R.O.W. (Goal, Reality, Options, Will) model [32] to address each participant’s capability, opportunity, and motivation to change sitting behaviour, utilising the COM-B model [33]; an approach used to explain sitting behaviour and used in previous physical activity intervention research [34]. Coaching sessions typically lasted between 15 and 30 min. Health coaches had a background in behaviour change and health psychology and received training specifically for this intervention [28].

#### Self-monitoring and prompt tools

Participants selected from wearables and apps that enabled self-monitoring of sitting, inactive time or computer use, and provided feedback and prompts to break up sitting [28]; see Supplementary Material 1. Participants chose a maximum of one tool from each of the following: (a) wearable device, (b) smartphone app, and (c) computer prompt software. Participants were required to choose at least one tool but were not required to choose a tool from every category and could switch tools during the intervention if they wished to. If a wearable device was chosen, these were sent out and returned by post.

### Control group

The control group continued to receive any usual diabetes healthcare as normal. Control participants were provided access to the online education session and the self-monitoring and prompt tools after the study had ended.

### Data collection

#### Trial feasibility, safety and acceptability

Participant recruitment, attrition and data measurement completion rates were used to evaluate trial feasibility. Safety was assessed in the context of serious adverse events. Semi-structured interviews (see below) explored acceptability of randomisation to the study groups and data collection procedures. A process evaluation questionnaire that was developed following Medical Research Council guidelines [35] and previous research [36] explored potential effects of the study measurements on participants’ behaviours [28].

#### Acceptability, experiences and adherence to the intervention

Intervention acceptability and experiences with the intervention were assessed as part of the process evaluation using semi-structured interviews conducted via video or telephone in a sub-sample of control participants, intervention participants, and health coaches. Convenience sampling was used, with participants invited to interview after 6-month data measurements. The aims of the qualitative analysis presented here were to explore the acceptability, feasibility and experiences of participating in the trial and the intervention [28]. The interview schedules are shown in Supplementary Material 2 and were informed by previous research [36].

Adherence was evaluated by recording the number of health coaching sessions attended and via process evaluation questionnaires at 3 and 6 months regarding online education session completion and the use of self-monitoring and prompt tools.

#### Study measurements

All participants completed the following measurements at baseline, then 3 and 6 months later. Information on COVID-19 circumstances were collected at each timepoint. Due to the COVID-19 pandemic, data collection sessions took place via video call. Equipment to take measurements was posted to participants. Participants were provided with a £10 shopping gift voucher for completing study measurements at each timepoint.

##### Sitting, standing and stepping

Mean daily sitting time was the proposed primary outcome for a definitive trial. The activPAL4 activity monitor (PAL Technologies, Glasgow, Scotland) was used to measure daily sitting, prolonged sitting (≥ 30-minute bouts), number of breaks in sitting, standing and steps. The device was attached to the anterior thigh by participants during a video call with a researcher and then worn for eight consecutive days. Participants were provided with a diary to record their sleep/wake times. Processing PAL v1.31 (University of Leicester, UK) was used to process and summarise event files created using PAL Batch (PAL Technologies, Glasgow, Scotland). Waking wear data was identified using a validated algorithm [37] within Processing PAL, which was cross-checked with diaries and manually corrected where appropriate. A valid day was considered as ≥ 10 h of wake time, < 95% of time spent in sitting, standing or stepping, and ≥ 1000 steps. Participants were included in the analysis if they had at least one valid wear day at all timepoints.

##### Waist circumference and physical function

Participants measured their waist circumference at the level of the umbilicus [28]. Informed by previous research in individuals with diabetes [38], the Short Physical Performance Battery (SPPB) was used to assess physical function [39]. This included rising from a chair, standing balance, and normal walking speed. Scores for each test and an overall SPPB summary score were calculated [39].

##### Psychological, wellbeing, musculoskeletal and sleep measures

Questionnaires were completed online using Qualtrics (Qualtrics, London, UK). Measures included the Chalder Fatigue Scale [40], Schwarzer and Renner Physical Exercise Self-Efficacy Scale (adapted to assess self-efficacy related to sitting less) [41], Generalised Self-Efficacy Scale [42], Cohen Perceived Stress Scale [43], Positive and Negative Affect Schedule [44], World Health Organization Five Well-Being Index [45], WHOQOL-BREF quality of life [46], Pittsburgh Sleep Quality Index [47] and Standardised Nordic Questionnaire to assess musculoskeletal symptoms [48].

### Data analysis

#### Trial feasibility and acceptability

Descriptive statistics were used to summarise participant eligibility, recruitment, retention and outcome measurement completion rates. Potential effects of the study measurements on behaviour were analysed using frequencies.

#### Qualitative analysis

**Fig. 1:**
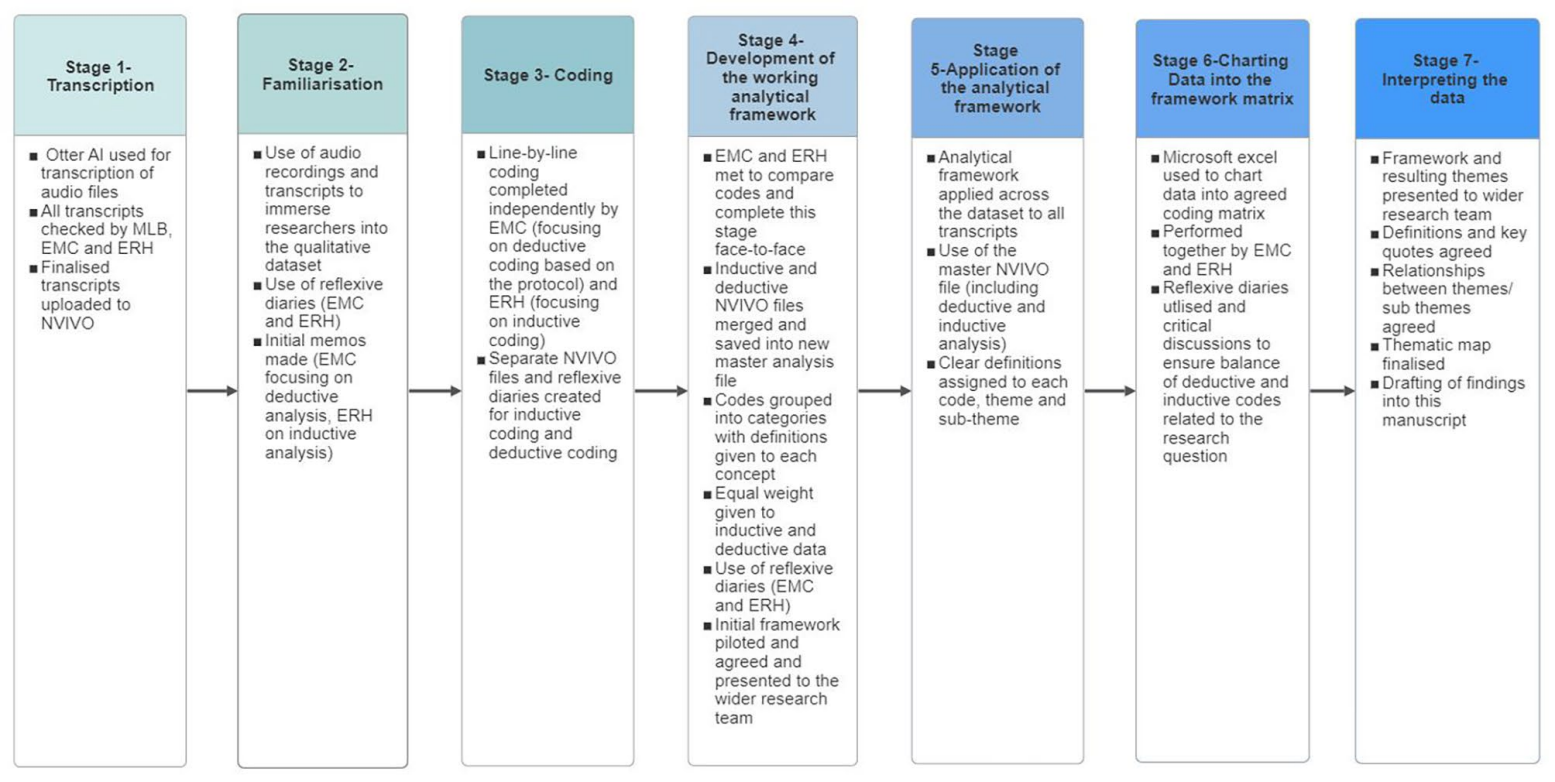
Implementation of the framework method for analysing the qualitative data

Interviews were conducted by MLB and recorded and transcribed verbatim using Otter AI (Otter.ai, Inc., Mountain View, CA, USA). Transcripts were checked manually by MLB, EMC and ERH, and imported into NVivo 12 (Lumivero, Denver, CO, USA) for analysis. Framework Method of Analysis [49] was used (which was a variation from the study protocol) to combine inductive and deductive analysis systematically, promote multi-professional analysis, align with the large data set (*n* = 30), and better address the aims of the nested qualitative study (Fig. 1). The initial stages (1 to 3) of the Framework Method were conducted independently by two researchers to identify inductive codes (ERH; based on the data) and deductive codes (EMC; based on the trial protocol), which were combined to develop the agreed ‘working analytical framework’ (stage 4) [49]. Reflexive journaling (EMC and ERH) and discussions within the qualitative research team (MLB, EMC, ERH, AMC), and wider research team, ensured reflexivity and rigour were maintained throughout the analysis (Fig. 1).

#### Potential to reduce daily sitting and improve proposed secondary outcomes in a definitive trial

The potential of the RESIT intervention for reducing daily sitting and changing secondary outcomes was explored using descriptive statistics (mean ± SD, frequency, counts and percentages). Data were analysed using Microsoft Excel v16.0 (Microsoft Corporation, Redmond, Washington, USA) and SPSS v26.0 (IBM Corp., Armonk, NY, USA). In line with good practice recommendations for pilot and feasibility studies [50], significance testing was not undertaken as formal sample size calculations had not been performed.

## Results

### Trial feasibility and safety

A total of 24 GP practices were approached, of which 19 (79%) participated. Study information was sent to 6,333 potentially eligible participants by these practices, of whom 95 (1.5%) expressed interest in taking part in the study. This was supplemented with other sources of recruitment, such as local Diabetes UK support groups. In total, 125 individuals expressed interest, and 70 (84%) were eligible to participate. The overall recruitment rate of individuals who were eligible was 67%. Table 1 shows the baseline demographic characteristics of the participants.

**Table 1:** Demographic characteristics of the participants at baseline

		Control (n = 35)	Intervention (n = 35)	All (n = 70)
Sex, n (%)	Male	13 (37%)	18 (51%)	31 (44%)
	Female	22 (63%)	17 (49%)	39 (56%)
Age (years), mean (SD)		55 (11)	60 (11)	58 (11)
Ethnicity, n (%)	Black, Asian and minority ethnic	22 (63%)	21 (60%)	43 (61%)
	White (any White background)	13 (37%)	14 (40%)	27 (39%)
Education, mean (SD)	Secondary school (e.g., high school)	8 (23%)	10 (29%)	18 (26%)
	Tertiary (e.g. university and above)	27 (77%)	25 (71%)	52 (74%)
Married/cohabiting, n (%)	Married/living as married	27 (77%)	17 (49%)	44 (63%)
	Single/separated/divorced/widowed	8 (23%)	18 (51%)	26 (37%)
Employment status, n (%)	Disabled	0 (0%)	2 (6%)	2 (3%)
	Employed full time	23 (66%)	12 (34%)	35 (50%)
	Employed part time	4 (11%)	4 (11%)	8 (11%)
	Retired	8 (23%)	14 (40%)	22 (31%)
	Student	0 (0%)	2 (6%)	2 (3%)
	Unemployed	0 (0%)	1 (3%)	1 (1%)
Had COVID prior to study start, n (%)	No	31 (89%)	34 (97%)	65 (93%)
	Yes	4 (11%)	1 (3%)	5 (7%)
Effects of the COVID-19 pandemic on work/life, n (%)	Currently shielding	6 (17%)	11 (31%)	17 (24%)
	Newly working from home	11 (31%)	8 (23%)	19 (27%)
	Unemployed or retired already	6 (17%)	10 (29%)	16 (23%)
	Lost their job	0 (0%)	1 (3%)	1 (1%)
	Been furloughed	6 (17%)	0 (0%)	6 (9%)
	Currently self-isolating	2 (6%)	2 (6%)	4 (6%)
	None of the above	8 (23%)	4 (11%)	12 (17%)
	Other	4 (11%)	6 (17%)	10 (14%)
Years living with type 2 diabetes*, mean (SD)		11 (8)	12 (11)	11 (9)

*Data on years living with type 2 diabetes was only available for 54 participants (n = 28 for usual care control group and n = 26 for intervention group)

**Fig. 2:**
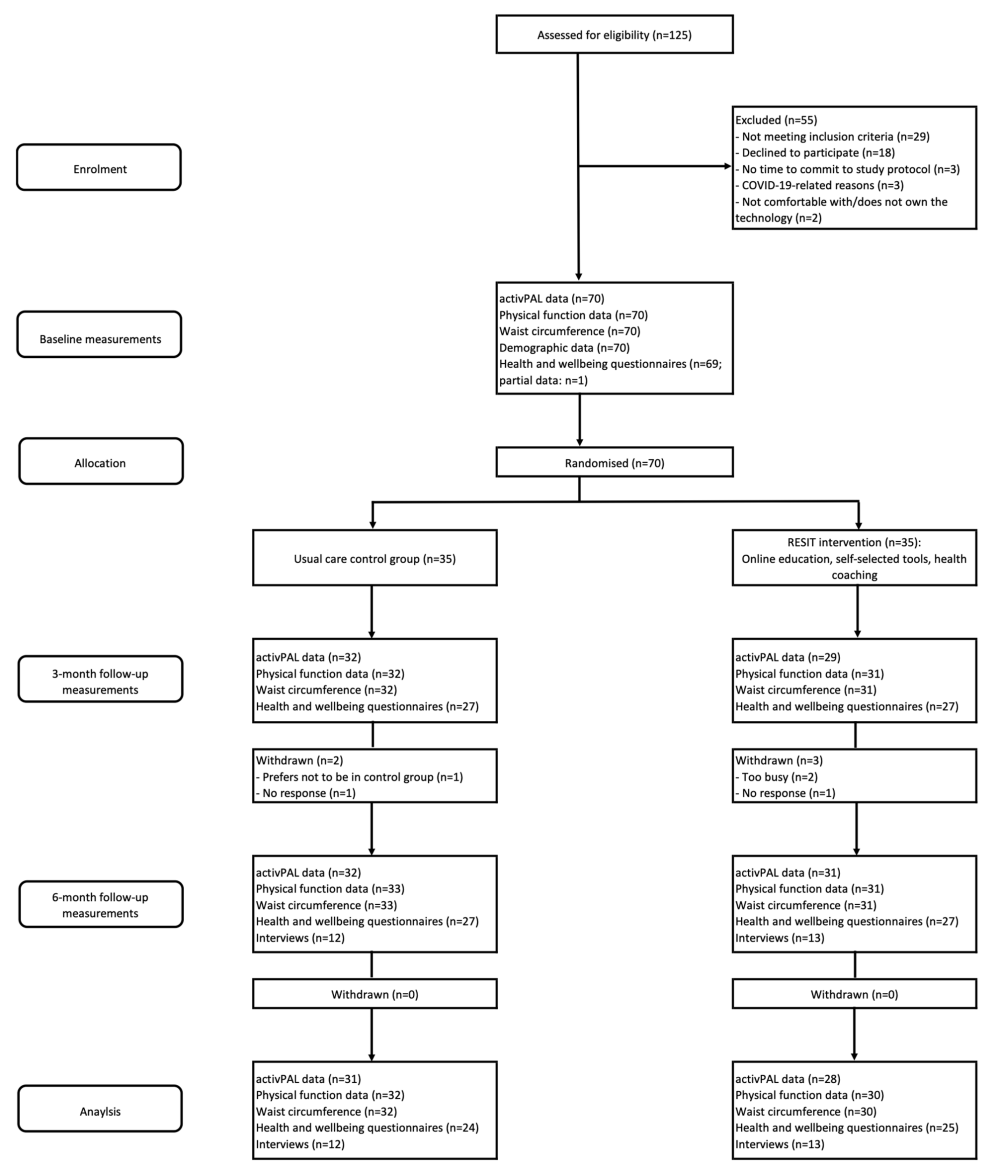
Flow of participants throughout the study

Participants were recruited from October 2020 to March 2021. Baseline data collection took place from January to March 2021. Measurements for the 3 and 6-month timepoints occurred from April to August 2021 and from August to November 2021, respectively. At the 3-month timepoint, 93% of the participants were retained (94% and 91% for control and intervention groups, respectively) with no further withdrawals at 6 months. The flow of participants throughout the study is shown in Fig. 2.

Completion rates for the intended primary outcome (daily sitting) were 100% for both groups at baseline. At 3 months, completion rates were 91% and 83%, and at 6 months were 91% and 89%, for the control and intervention groups, respectively. This was based on providing ≥ 1 valid day of activPAL data (see Table 2). Data completion rates for the secondary outcomes are shown in Table 2. There were no serious adverse events reported that related to the study procedures.

**Table 2:** Completion rates for the study measurements at each time point. Data shown as N (%)

	Baseline		3 months		6 months		All time points	
	Control (*N* = 35)	Intervention (*N* = 35)	Control (*N* = 33)	Intervention (*N* = 32)	Control (*N* = 33)	Intervention (*N* = 32)	Control	Intervention
activPAL data								
≥ 1 valid day	35 (100%)	35 (100%)	32 (91%)	29 (83%)	32 (91%)	31 (89%)	31 (89%)	28 (80%)
≥ 2 valid days	34 (97%)	35 (100%)	32 (91%)	29 (83%)	32 (91%)	30 (86%)	30 (86%)	27 (77%)
≥ 3 valid days	33 (94%)	34 (97%)	32 (91%)	29 (83%)	32 (91%)	29 (83%)	29 (83%)	26 (74%)
≥ 4 valid days	32 (91%)	33 (94%)	32 (91%)	29 (83%)	32 (91%)	29 (83%)	29 (83%)	25 (71%)
≥ 5 valid days	31 (89%)	33 (94%)	30 (86%)	28 (80%)	32(91%)	27 (77%)	27 (77%)	24 (69%)
Short physical performance battery	35 (100%)	35 (100%)	32 (91%)	31 (89%)	33 (94%)	31 (89%)	32 (91%)	30 (86%)
Waist circumference	35 (100%)	35 (100%)	32 (91%)	31 (89%)	33 (94%)	31 (89%)	32 (91%)	30 (86%)
Health and wellbeing questionnaires	35 (100%)	34 (97%)	27 (77%)	27 (77%)	27 (77%)	27 (77%)	24 (69%)	25 (71%)

Data completion rates were calculated as the number of complete datasets for each outcome measure / number of participants enrolled at baseline x 100

### Effects of study measurements on behaviour

Of the participants who completed the process evaluation questionnaire, 77% and 73% of the intervention participants agreed or strongly agreed that taking part in the study measurements motivated them to change aspects of their behaviour at 3 and 6 months, respectively. This was lower in the control group with 29% and 41% agreeing or strongly agreeing at 3 and 6 months, respectively. With respect to follow-up study measurements motivating participants to make changes to their sitting time, 72% and 78% of intervention participants agreed or strongly agreed at 3 and 6 months. In the control group, 33% and 41% agreed or strongly agreed at 3 and 6 months (Supplementary Material 3).

### Nested qualitative results

**Fig. 3:**
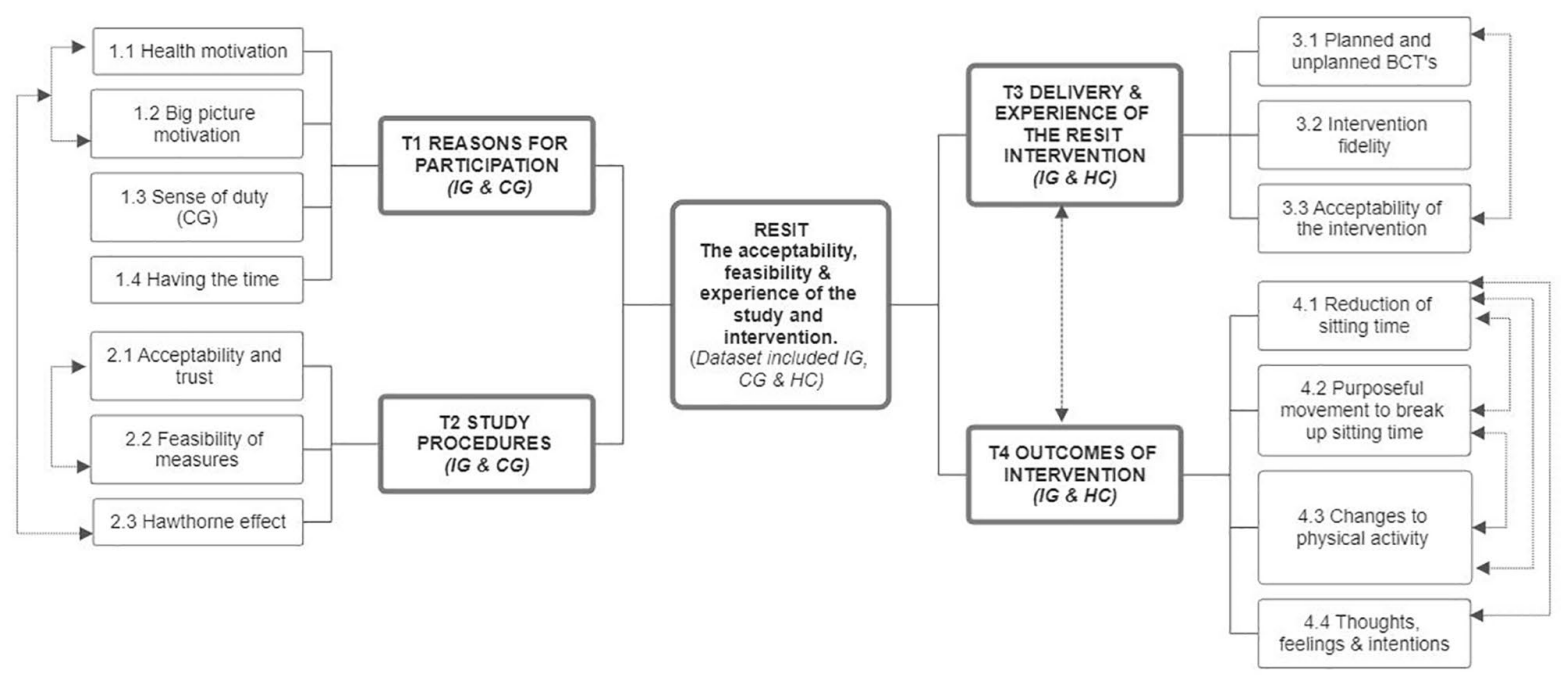
Thematic map of the RESIT study nested qualitative analysis. CG, control group; IG, intervention group; HC, health coaches; T, theme; BCTs, behaviour change techniques

A subset of 25 participants (*n* = 12 control and *n* = 13 intervention participants) and all health coaches (*n* = 5) were interviewed (sample characteristics are shown in Supplementary Material 4). Interviews with participants and health coaches lasted 37 ± 14 min (range 14–72) and 32 ± 8 (range 22-43), respectively. A thematic map of the themes and subthemes of the nested qualitative data is shown in Fig. 3. A total of four themes were constructed. This paper focuses on three themes (reasons for participation; study procedures; delivery and experience of the RESIT intervention) and sub-themes 1.3, 2.1 to 2.3 and 3.1 to 3.3 that align with the feasibility and acceptability aims reported in this paper (Table 3 shows the specific themes, definitions and illustrative quotes).

**Table 3:** Themes, definitions and illustrative quotes for the nested qualitative analysis

Theme and subtheme	Definition	Illustrative quotes (quote, participant ID, dataset i.e., IG, CG or HC)
Theme 1. Reasons for participation*		
1.3 Sense of duty	This subtheme includes CG participants understanding of the importance of a CG and the commitment they made to the study. They expressed a sense of responsibility.	*‘This work might lead to something that gets implemented by the NHS, which could benefit millions of people…So, you know, it would be churlish not to continue with the study’ (RS60, CG when asked on thoughts when found out they were randomised to the CG)* *‘Well, I made a commitment. I must admit that I was disappointment to be in the control group, because I wanted to be, you know, more active I wanted to be, you know, more part of, you know the, you know the science involved, but I’d made a commitment to you that I would do it…I’d made a commitment, you know, it’s not very onerous is it?’ (RS23, CG)* *‘Well, I mean, I was disappointed because you know, part of my reasons for signing on was to see what it was all about and get it…if you haven’t got a control group, you haven’t got a study. So and you know, this work might lead to something that gets implemented by the NHS’ (RS60, CG)*
*Theme 2. Study procedures*		
2.1 Acceptability and trust	This subtheme includes participants’ voices (CG and IG) regarding the acceptability and trust of study procedures.	*‘How do you make a diabetic study if you don’t have any impact on the sugar levels?…I think It’s irrelevant. It doesn’t matter. If you don’t, if you don’t know what your sugar levels are’ (RS25, CG, the importance of diabetic specific measurement)* *‘Measuring your own waist circumference. I mean, anybody measuring my waist circumference to me is, is always going to be out’ (RS60, CG, challenges with performing own waist circumference measurement)* *‘The walking and saying ‘stop’ I never quite understood that one, because I didn’t, I never thought it was that long as length of space’ (RS07, IG, questioning the validity of the 8-foot walk test within the SPPB)*
2.2 Feasibility of measures	This subtheme explores the participants perceptions of the feasibility of the measures (CG and IG) i.e., can the study procedures/measures be carried out?	*‘I liked having the instruction sheet. So, I knew on the first session what I was going to have to do’ (RS46, CG)* *‘Yeah, it’s the plaster bit you know where you you cover it and you stick it on your thigh? Yeah. Somehow. It wasn’t so much of a allergic reaction. Such. Yeah. It. I think I started to itch a little bit’ (RS30, CG)* *‘Apart from having to be careful when I was in the shower. I didn’t have an issue with it. You know, and even then the the way that it was connected to my leg or affixed to my leg was quite waterproof so i It wasn’t an issue for me’ (RS04, IG)* *‘it’s not very onerous is it? Every couple of months, you know, do a couple of exercises and put a tab [activPAL] on your knee I mean it’s on your leg. It’s not very onerous’ (RS23, CG)*
2.3 Hawthorne effect	Participants in the CG, and some in the IG, mentioned changes to their behaviour (sitting and physical activity) in response to measurement and observations i.e. a Hawthorne effect. In addition, there was one example of contamination.	*‘Every time I was standing up, either when I was in the garden or doing the washing up, or wherever, going for a walk or whatever, psychologically in my brain, I was thinking, “that’s a good thing,” because this study is all about, you know, measuring, you know, how often you stand up, whatever, you know, cutting [did enough] time that you spend sitting down, so psychologically, I do think that being, having had to chat with you at the start of the, of the, of the course, of the of the study, you know, and when you explained what it was all about, that had an impact on me’ (RS23, CG, Hawthorne effect)* *‘A little bit in the sense that, you know, if I’m watching TV, literally, you know, if we’re having an early dinner, and I’ve got to have a time in between. So when I’m sitting down to watch TV, I would, you know, do the steps. Just get up and just march’ (RS30, CG, Hawthorne effect- creating own intervention)* *‘Yeah ((overlaps)) So, so I understood, but if I didn’t understand would I have done it? I dunno? But I think Mum was a good reminder because mum was having stuff so that was my kind of- So remember, I’ve got this other reminder going on. ((phone rings)) Sorry, Marsha, now my phone is going on’ (RS09, CG- Potential Contamination-Mother in the RESIT IG)* *‘I felt a bit of like, oh for the next one. I want to be speedier, I want to be going to walk more I want to be thinner. (laugh) I was motivated for the first one [measurement]. But when I like I said, I think when I didn’t get any feedback, I just felt- oh well, I’m not going to know so whatever. It’s just, do whatever’ (RS14, IG, Hawthorne effect– motivation to improve measurements)* ***Contrasting quote*** *‘I don’t remember the measurements. And I didn’t. I didn’t pay attention to it. I felt maybe it’s not as important’ (RS25, CG- contrasting quote on Hawthorne effect)*
*Theme 3. Delivery and experience of the RESIT intervention*		
3.1 Planned and unplanned BCTS	This subtheme includes the active ingredients (BCTs) the intervention participants (IG) and HC’s acknowledged. This includes those in the protocol and any additional BCT’s.	*‘The only thing I did do if I was watching a program and I knew, I watch a lot of series, and they would normally be about 45 min long. And and I’d know that I’ve watched, before I come to the end, and I said, ‘time to get up and go and have little wander around’. That’s basically all I added to mine, yeah’ (RS63, IG, BCT 1.2 Problem solving)* *‘I felt like I had something new to tell her every time a new goal I hit, something that I was doing that I could I could speak to her about? And it was almost pleasing her. (laugh)’ (RS14, IG, BCT 1.1 goal setting (behaviour); BCT 1.5 review behaviour goals)* *‘That was the eye opener, I think that was the you know, the little movements that make a difference’ (RS14, IG, BCT 2.2 feedback on behaviour)* *‘Yeah, he was very good listener in the beginning, and then he will just um tell me that, okay, instead, why don’t you try this way? And just give it a go and see how it feels, you know? He was always giving me the healthy choice and suggestions’ (RS68, IG, BCT 3.1 social support [unspecified])* *‘what, what the RESIT did was to really, really reignite in my mind This this television program about the poisons that build up If you’re not, I won’t say continually active, but if you’re sat down, you shouldn’t be sat down for prolonged periods of time’ (RS42, IG, BCT 5.1 information about health consequences)* *‘Cos if you’re wearing a watch, you’re doing the steps, it’s recording your sleep, your heartbeat, etc, etc. And it does tell you you’ve been sitting for too long so you can stand up and you know, walk about for a few minutes, then that’s really helpful. It does occasionally but then it goes quiet for maybe a day or two and doesn’t do anything. Then all of a sudden tell me I’ve been sitting for too long. And I think, ‘whoa, I’ve been sitting for two days’. You’ve got to see the funny part of this as well ((laughs))’ (RS63, IG, BCT 2.3 self-monitoring of behaviour; BCT 7.1 prompts/cues)* *‘I think the most obvious one, I guess, is the Stand Up! app. Oh yeah, I think. I wasn’t aware of it as an app as such but I was aware of the need to get up every 45 min for five minutes or what have you. But in having the app and using it has made a difference. And, you know, I got to be honest, there are days when, you know, even though the app tells me to get up you can’t because of work demands, but at least it does allow you to start doing some of those little things easily. So that helps’ (RS22, IG, BCT 7.1 prompts/cues)* *‘I did quite quite often emphasize that it doesn’t have to be you don’t have to be doing formal exercise or activity doesn’t have to be a walk, it can just be like taking a break. A lot of the time is taking a break from your desk at work and getting up to go to the loo or getting a coffee or chatting to a colleague things like that. So those are all things to break up their sitting time. So, a lot of them found it kind of challenging at first too, because you’re so used to just sitting down and working away all day. But the more you kind of get these habits built into place, the more they become quite easy to follow up with after time’ (HC1, BCT 15.1 verbal persuasion about capacity)* *‘It was like the motivation kick for those during that sessions, you know, give chance to express feelings. how we feel. Yeah, it was good’ (RS47, IG, BCT 3.3 = social support [emotional] - additional BCT not specified in the protocol)*
3.2 Intervention fidelity	This subtheme looks at intervention fidelity i.e., was the intervention carried out the way it was intended?	*‘So actually, on that note, I think only one participant decided that they only wanted three calls. And they didn’t need the fourth one. So they’re just going through that. So that’s fine. And yeah, I didn’t know. On the coach side. I was like, Do you guys want me to definitely do the fourth those that and be so I think I didn’t do about that. But yeah, we just did three and that was fine’ (HC4)* *‘I’m not sure I’ve done any of that have I?’ (RS44, IG when asked about health coaching sessions).* *‘Same thing, the fear of technology, I don’t want to learn new, new new technology things. Because the brain is so scarred up with so much new things. And it’s so deep and variety of work. I’m getting involved. And now it feels like over in the brain that I don’t want more information’ (RS47, IG- when asked why they did not engage with the apps)*
3.3 Acceptability of the intervention	This subtheme theme includes acceptability of the RESIT intervention components including the online education session, the health coaching sessions, and the apps and wearables. There is a link to the BCTs as these were embedded in all aspects of the RESIT intervention.	***Online education session*** *‘I can’t remember. But if I go on website now, then I can perhaps understand a bit more. But I can’t remember right now’ (RS47, when asked about online education package)* ***Health coaching*** *‘So those are all things to break up their sitting time. So a lot of them found it kind of challenging at first too, because you’re so used to just sitting down and working away all day. But the more you kind of get these habits built into place, the more they become quite easy to follow up with after time’ (HC1)* ***Wearables and apps*** *‘So the stand up! App, as an example, I’m still using it now. The app’ (RS22, IG)* *‘If I was sitting for too long, the reminder was good. But if I had got up, then I thought Oh not sure about this, I don’t know’ (RS11, IG)*

Note. * Theme 1 (1.1, 1.2) and Theme 4 are presented elsewhere as part of the study’s process evaluation analysis. IG, intervention group; HC, health coach; CG, control group; BCTs, behaviour change techniques; RESIT, REgulate your SItting Time

#### Theme 1– Reasons for participation

##### Subtheme 1.3 - Sense of duty (control group)

Control group participants were aware of the importance of their involvement in the study and the wider implications of taking part, such as the potential benefit to others living with T2DM. Despite this understanding, many suggested they were ‘disappointed’ to have been allocated to the control group because of wanting to “see what it was all about”. Nonetheless, the control group participants expressed a sense of responsibility to the study, often explaining that they continued with the study due to having made the ‘commitment’ to participate.

#### Theme 2 - Study procedures

##### Subtheme 2.1 - Acceptability and trust

The acceptability and feasibility of the study procedures was discussed by participants, as well as a potential Hawthorne effect in response to taking part in the study measurements. Participants regularly expressed the importance for “sugar levels” (e.g., HbA1c) to be measured in future studies as it would provide more meaningful data about the impact of the intervention on their diabetes. Similarly, there was some concern from participants regarding the accuracy of taking study measurements themselves.

##### Subtheme 2.2 - Feasibility of measures

Despite some perceived limitations with the study measures, participants considered the trial to be feasible overall with study procedures being described as “not very onerous”. Some minor discomfort from wearing the activPAL, such as irritation or itching, was expressed by some participants. However, this was also perceived by participants to be managed and resolved quickly by the research team.

##### Subtheme 2.3 - Hawthorne effect

When discussing study procedures, it was found that most control participants and some intervention participants expressed changing their behaviour in response to being measured or observed. This often occurred through making efforts to reduce sitting or increase their physical activity levels. One control group participant mentioned that a family member was in the intervention group. All other control group participants that were interviewed reported no contact with the intervention group.

#### Theme 3 - Delivery and experience of the RESIT intervention

##### Subtheme 3.1 - Planned and unplanned BCTs

The intervention participants and health coaches perceived there to be several BCTs experienced that were intended to be in the intervention, namely BCTs 1.1 (goal setting [behaviour]), 1.2 (problem solving), 1.5 (review behaviour goals), 2.2 (feedback on behaviour), 2.3 (self-monitoring of behaviour), 3.1 (social support [unspecified]), 5.1 (information about health consequences), 7.1 (prompts/cues) and 15.1 (verbal persuasion about capability) [51]. Qualitative analysis revealed an additional BCT (3.3 social support [emotional]). Overall, intervention participants valued prompts and cues (BCT 7.1) and social support (unspecified) (BCT 3.1) when engaging with the health coaches to reduce sitting. Some participants chose to use the apps, others set timers or ‘clock watched’, and some used physical reminders such as advertisement breaks when watching TV to prompt them to reduce and break up sitting.

##### Subtheme 3.2 - Intervention fidelity

Most intervention participants expressed value in the health coaching sessions, although one participant did not recall them. Participants found the self-monitoring and prompt tools useful for making them aware that they had been sitting for too long and reminding them to move regularly. Some participants reported variable engagement with the self-monitoring and prompt tools due to fear or limited knowledge of technology, and personal preference.

##### Subtheme 3.3 - Acceptability of the intervention

The prompts and cues delivered through the wearables and apps were viewed as a useful component of the RESIT intervention. At times, participants struggled to recall the online education session in detail, but remembered the overall message and the importance of reducing their sitting for managing their health and T2DM. One participant reported struggling to build rapport with their health coach. All other participants valued the problem solving and ability for the health coaches to support them with personalising their sitting behaviour goals. This was echoed by the health coaches’ encounters, who valued supporting participants during the intervention to overcome challenges and meet their goals. The health coaches also valued the training provided to them during the study, reporting that the application of the motivational interviewing, COM-B model and G.R.O.W training helped them engage participants with the intervention.

### Intervention engagement

The process evaluation questionnaire exploring intervention engagement was completed by *n* = 22 (63%) intervention participants at each of the 3 and 6-month timepoints. The online education session was, according to self-report, fully completed by 82% (*n* = 18) of intervention participants who completed the survey, and partially completed by 14% (*n* = 3). In the first 3 months, 82% (*n* = 18) of intervention participants reported using a wearable device, 77% (*n* = 17) reported using a smartphone app, and 18% (*n* = 4) reported using computer prompt software for reducing and breaking up sitting. At 6 months, 68% (*n* = 15) of participants reported using a wearable device, 55% (*n* = 12) reported using a smartphone app, and two participants (10%) reported using computer prompt software. Participants who used a wearable reported high usage in the first 3 months, with 74% using it at least 5 days/week. In months 3 to 6, 80% of participants who used a wearable reported using it at least 5 days/week. There was attendance at 81% of the 140 health coaching sessions that were available to the intervention participants during the study, with each participant attending at least two of the four sessions offered to them.

### Potential of the intervention to reduce daily sitting and improve secondary outcomes

At 3 months, the intervention and control groups reduced their daily sitting by 30.9 ± 87.2 min/day (a 5% reduction of waking wear time) and 4.4 ± 99.5 min/day (a 2% reduction of waking wear time), respectively (Table 4). Both groups had reduced their sitting by a similar volume at 6 months relative to baseline (-22.2 ± 82.5 and − 23.7 ± 85.2 min/day for the intervention and control groups, respectively), equal to a 3% reduction of waking wear time. Prolonged sitting was reduced by 51.9 ± 104.1 min/day in the intervention participants and 16.5 ± 108.8 min/day in the controls at 3 months. At 6 months, there was a 42.5 ± 99.3 min/day reduction in the intervention group and 43.6 ± 102.4 min/day reduction in the control group. The 5% reduction in daily sitting at 3 months for the intervention participants was replaced by standing (3%; 31.8 ± 60.7 min/day) and stepping (2%; 17.0 ± 19.1 min/day). At 6 months, standing replaced 1% (11.0 ± 46.1 and 16.8 ± 64.1 min/day for the intervention and control groups, respectively) and stepping replaced 2% (14.5 ± 19.6 and 19.4 ± 28.7 min/day for the intervention and control groups, respectively) of the 3% reduction in daily sitting. The number of breaks in sitting (sit-to-upright transitions) was higher by 3.8 ± 12.2 per day at 3 months in the intervention group, which was maintained at 6 months (3.5 ± 12.3 per day); there was a 1.5 ± 12.2 per day and 1.7 ± 8.9 per day increase at 3 and 6 months, respectively, in the controls.

**Table 4:** Descriptive statistics for the activPAL variables

	Baseline		3 months		6 months		Change (baseline to 3 months)		Change (baseline to 6 months)	
	Control	Intervention	Control	Intervention	Control	Intervention	Control	Intervention	Control	Intervention
**Variable**	(*n* = 31)	(*n* = 28)	(*n* = 31)	(*n* = 28)	(*n* = 31)	(*n* = 28)	(*n* = 31)	(*n* = 28)	(*n* = 31)	(*n* = 28)
Waking wear time (minutes/day)	934.9	909.0	951.5	926.8	947.4	912.3	16.6	17.9	12.6	3.4
	(58.3)	(69.4)	(51.2)	(72.6)	(50.8)	(61.2)	(49.2)	(58.0)	(47.2)	(59.5)
Daily sitting (minutes)	665.6	642.9	661.2	611.9	642.0	620.7	-4.4	-30.9	-23.7	-22.2
	(102.9)	(114.4)	(93.0)	(116.8)	(86.1)	(93.1)	(99.5)	(87.2)	(85.2)	(82.5)
Daily standing (minutes)	193.2	195.5	196.4	227.3	210.0	206.5	3.2	31.8	16.8	11.0
	(77.6)	(75.4)	(70.5)	(85.3)	(69.8)	(67.2)	(74.2)	(60.7)	(64.1)	(46.1)
Daily stepping (minutes)	76.1	70.6	93.9	87.6	95.5	85.1	17.8	17.0	19.4	14.5
	(34.4)	(33.1)	(38.4)	(32.5)	(37.7)	(36.0)	(26.8)	(19.1)	(28.7)	(19.6)
Steps per day	6044	5560	7655	7081	7847	6978	1612	1521	1803	1418
	(3225)	(2934)	(3543)	(2954)	(3681)	(3549)	(2431)	(1517)	(2922)	(1961)
Percentage of wear time spent sitting	71	71	69	66	68	68	-2	-5	-3	-3
	[9]	[11]	[8]	[11]	[8]	[9]	[9]	[8]	[8]	[7]
Percentage of wear time spent in sitting bouts ≥ 30 min	45	46	43	39	40	41	-3	-6	-5	-5
	[12]	[13]	[10]	[12]	[11]	[10]	[11]	[11]	[10]	[9]
Percentage of total sitting time spent in bouts ≥ 30 min	63	64	61	59	59	60	-2	-5	-4	-4
	[12]	[12]	[10]	[13]	[12]	[10]	[10]	[10]	[9]	[9]
Percentage of wear time spent in sitting bouts 0–30 min	25.9	25.1	26.7	26.8	27.6	27.1	0.8	1.7	1.6	2.0
	(8.5)	(7.6)	(6.7)	(8.6)	(7.9)	(6.8)	(6.1)	(5.3)	(5.5)	(5.5)
Percentage of wear time spent standing	(8.5)	(7.6)	(6.7)	(8.6)	(7.9)	(6.8)	(6.1)	(5.3)	(5.5)	(5.5)
	[8]	[9]	[8]	[9]	[7]	[7]	[8]	[7]	[6]	[6]
Percentage of wear time spent stepping	8	8	10	9	10	9	2	2	2	2
	[4]	[4]	[4]	[4]	[4]	[4]	[3]	[2]	[3]	[2]
Number of sit-upright transitions	42.8	41.7	44.3	45.5	44.5	45.2	1.5	3.8	1.7	3.5
	(14.0)	(11.5)	(12.8)	(16.1)	(13.8)	(14.4)	(12.2)	(12.2)	(8.9)	(12.3)
Time in sitting bouts ≥ 30 min (minutes)	423.0	415.5	406.5	363.5	379.3	372.9	-16.5	-51.9	-43.6	-42.5
	(121.9)	(125.8)	(104.0)	(117.4)	(101.7)	(91.4)	(108.8)	(104.1)	(102.4)	(99.3)
Time in sitting bouts 0–30 min (minutes)	242.6	227.4	254.7	248.4	262.6	247.7	12.1	21.0	20.0	20.4
	(81.1)	(67.1)	(69.4)	(3.2)	(83.2)	(63.6)	(57.1)	(54.5)	(49.3)	(50.7)

Data are presented as mean (SD) for participants who provided at least one valid wear day at all timepoints

The intervention group had a 2.3 ± 13.8 and 3.9 ± 13.5 cm lower waist circumference at 3 and 6 months, respectively, compared with baseline (Supplementary Material 5). In the control group, waist circumference was unchanged at 3 months (0.1 ± 0.5 cm) and 1.5 ± 5.4 cm lower at 6 months. There were no apparent changes in physical function in either group (Supplementary Material 5). Health, wellbeing, psychological and sleep questionnaire outcome data can be seen in Supplementary Material 5. There appeared to be improvements in physical, psychological, social relationships and environment quality of life domains at 3 and 6 months in the intervention group, but not in the controls. Improvements were also seen in self-efficacy related to sitting less, wellbeing and negative affect. The control group’s scores on these outcomes did not appear to improve during the study.

## Discussion

The findings of this study demonstrate, for the first time, the feasibility of delivering and evaluating a tailored remote intervention to reduce and breaking up sitting in ambulatory adults living with T2DM. Recruitment of participants into the study was achieved within an acceptable timeframe alongside sufficient recruitment, retention and data completion rates. Qualitative results revealed that control group participants felt a ‘sense of duty’ to continue with the study despite being randomised to that study arm. Overall, participants felt the data collection visits were straightforward and easy to follow. However, some participants suggested the need for diabetes specific outcomes. The intervention was deemed acceptable and feasible, with prompts, cues and support from health coaches with goal setting suggested as particularly valuable components of the intervention.

The number of potentially eligible patients who expressed interest in taking part in the study was low relative to the number of patients approached by GP practices. A similarly low response rate of 4% to email invitations sent by a local Diabetes Australia branch was reported in a study evaluating a 12-week web-based physical activity intervention for adults with T2DM [52]. Recruiting patients through GP practices is often challenging in clinical trials due to factors such as time constraints, low remuneration and GPs forgetting to approach patients [53]. The present study had the additional challenge of patients not having face-to-face appointments and increased primary care workload due to the COVID-19 pandemic that may have impacted the amount of support GPs could provide for participant recruitment. Despite the relatively low interest from patients in this study, eligibility and recruitment rates were sufficiently high to achieve the target sample size. Furthermore, an inclusive sample that was representative of the general T2DM population in the UK was recruited [54], reflected by the high proportion of Black, Asian and minority ethnic participants, and 56% being female. This suggests that the recruitment strategies employed are likely to be suitable in a definitive randomised controlled trial (RCT) that seeks to recruit a representative sample of individuals with T2DM.

The trial had a high retention rate with only 7% (*n* = 5) of participants withdrawing from the study by 6 months. A systematic review reported a wide range of dropout rates (0 to 40%) for controlled studies evaluating structured exercise and physical activity advice interventions in people with T2DM [55]. There may be several factors affecting retention, such as the nature and burden of intervention and data collection protocols. In a web-based physical activity intervention for adults with T2DM, there was a dropout rate in the control and intervention groups of 51% and 45%, respectively, at 36 weeks [52]. All recruitment, intervention and data collection activities took place remotely in the present study, partly due to the COVID-19 pandemic. This may have addressed issues of compliance by reducing the burden associated with taking study measurements, such as travel and time. There was high compliance with the activPAL measure (daily sitting) at levels similar to previous intervention studies that used the activPAL device in office workers and adults at risk of T2DM [36, 56, 57]. A number of originally planned outcome measures could not be included due to the remote procedures, such as biochemical markers. Participants voiced the importance of including diabetes related measures, such as HbA1c, in future studies. The choice of diabetes outcomes can be informed by unified international recommendations on standard person-centred outcomes for diabetes [58]. There were also potential limitations with conducting timed physical function measures via video call. Based on internet connectivity, audio-visual delays could have been present that would have affected the times recorded. In addition, participants questioned the accuracy and relevance of the normal walking speed test due to it being over such a short distance. The findings of this study demonstrate the benefits of evaluating behaviour change interventions remotely, but these should be weighed up against potential drawbacks to ensure the intended outcomes are not compromised and participant trust is maintained.

The remotely delivered intervention in the present study appeared to address issues with adherence reported in previous remote web-based interventions with sedentary individuals and people with T2DM, despite high levels of satisfaction [52, 59, 60]. The improved adherence in this study could be due to participants being able to tailor the intervention to their own preferences with a choice of self-monitoring and prompt tools. Self-monitoring and prompts/cues have been identified as promising BCTs in interventions targeting reductions in sitting [20, 21]. The participants in this intervention reported using a range of tools to prompt breaking up sitting. The use of a wrist-worn device for self-monitoring inactive time and steps, and receiving alerts to get up and move regularly, was viewed as the most memorable and enjoyable part of the intervention. Wearable devices that deliver these BCTs have been used in previous interventions that have reduced sitting in office workers [61] and cancer survivors [62]. Some participants used smartphone or computer apps to prompt breaks in sitting, which have been reported as acceptable and effective in short-term interventions in the general population [63], office workers [61], and people with T2DM [22]. However, others used timers, clock watching, and functional prompts such as TV advertisement breaks, as reminders to break up sitting. Enabling individuals to personalise the way in which they engage in an intervention to reduce and break up sitting is, therefore, acceptable and valued by individuals with T2DM.

Similar to previous studies [60], the health coach support in the RESIT intervention may have been a key component supporting adherence. Indeed, participants reported high acceptability and value of these supportive sessions. The individualised problem-solving that was facilitated by the health coaching sessions was seen as a valuable intervention component, in line with previous research [56, 64]. During the follow-up interviews some participants demonstrated difficulty recalling the online education component of the RESIT intervention in detail, despite nearly all participants reporting completing it fully or partially. The key message from the online education session to reduce and break up sitting, though, was well remembered. A similar iteration of the online education session was used in an intervention (SMART Work & Life) that led to reductions in occupational sitting in office workers over 12 months [65]. There were similar levels of engagement to the present study with 81% of participants completing the session [66]. The SMART Work & Life participants appeared to recall the online education session to a better degree than in the RESIT intervention, but some did suggest that an in-person education session could be preferable and make them more memorable [67]. An online education session may, therefore, be warranted as a strategy for communicating key sedentary behaviour intervention messages. It may be appropriate for some in-person education to also be delivered as part of such interventions and this should be explored with the target population group during the intervention development phase.

The qualitative analysis found that some control participants created their own strategies to reduce sitting, knowing that this was an aim of the study. Changing behaviour in response to sedentary behaviour and physical activity measurement has been reported [68]. In this study, both control and, to a greater extent intervention participants, reported that taking part in the study measurements motivated them to change their sitting time. It could be argued, therefore, that the control group’s reduction in sitting at 6 months may not be due to a potential Hawthorne effect because similar, or even greater, changes in sitting would be expected in response to this in intervention participants. As part of the process evaluation, some control participants reported taking up exercise classes and joining the gym in the final 3 months of the study, which may have been motivated by relaxation of COVID-19 lockdown restrictions. Special measures to avoid behaviour change in a control group may, therefore, not be required in future evaluations of the RESIT intervention if it is not impacted by government restrictions requiring individuals to isolate at home or distance themselves from others.

This study extends the findings from previous studies with office workers by demonstrating that an intervention involving online education, self-monitoring and prompt tools, and health coaching for behavioural support has potential for reducing daily and prolonged sitting in people with T2DM [36, 61, 65]. The RESIT intervention also showed potential for improving waist circumference, wellbeing, negative affect, quality of life and self-efficacy related to sitting less. Improvements in sitting, prolonged sitting, sit-to-upright transitions, and health and wellbeing measures were observed in a feasibility evaluation of an 8-week smartphone app intervention (comprising self-monitoring, prompts/alerts and goal setting) in individuals with T2DM [22]. The present study extends knowledge by demonstrating the potential for improving sitting, health and wellbeing outcomes over a longer time period. Reductions in daily sitting and waist circumference observed in the intervention group could be clinically relevant in light of evidence that replacing 30 min/day of sitting with light-intensity physical activity is associated with improvements in insulin sensitivity and cardiometabolic biomarkers [69, 70]. There is also a significantly higher risk of cardiovascular disease for each 1 cm increase in waist circumference [71]. The effectiveness of interventions to reduce and break up sitting in individuals with T2DM now requires evaluation in fully powered RCT designs.

The strengths of this study include the mixed methods design to provide an in-depth understanding of the trial’s feasibility and acceptability. This has informed the suitability of procedures and refinements to the intervention for a future definitive trial. The characteristics of the participants were largely representative of the general T2DM population regarding age, sex, and ethnicity [54]. The remote nature of the intervention may also increase accessibility to patients. These factors suggest that the trial is likely to be feasible on a larger scale across various geographic locations and diverse participants. Due to the remote nature of the study, this may have biased the sample to individuals with better access and competence with digital technology. Strategies to promote digital inclusion should be considered in future studies of this nature. Participants were also required to undertake study measurements at home, which may have affected their precision. This limitation would be overcome with the conduct of a future study that is not impacted by pandemic-related restrictions. Another limitation was that the interviews with intervention participants did not specifically explore the presence of BCTs that were planned to be delivered within the RESIT intervention. This may be the reason that a number of planned BCTs were not identified through the qualitative analysis.

## Conclusions

This study demonstrated the feasibility and acceptability of delivering and evaluating a tailored remote intervention to reduce and break up sitting in people with T2DM. The trial and intervention were deemed acceptable. The health coaching and choice of wearables and apps for self-monitoring and prompts/cues were particularly valued. Future studies should consider design features to minimise any potential Hawthorne effect and the inclusion of outcomes that are meaningful to people living with T2DM. There was potential for the intervention to reduce daily and prolonged sitting, at least in the short-term, as well as improving health and wellbeing outcomes. A definitive trial, informed by these findings, is warranted to evaluate the effectiveness of the intervention to support the management of T2DM.

### Electronic supplementary material

Below is the link to the electronic supplementary material.

Supplementary Material 1

Supplementary Material 2

Supplementary Material 3

Supplementary Material 4

Supplementary Material 5

## Data Availability

The datasets supporting the conclusions of this article are available in Figshare, 10.17633/rd.brunel.25144490. The raw qualitative data (transcripts) are not publicly available due to privacy restrictions. Further detail on the qualitative data and analysis that supports the findings of this study are available upon request to the corresponding author.

## Abbreviations

BCT: behaviour change technique
COM-B: capability, opportunity, and motivation - behaviour
GP: General Practitioner
G.R.O.W.: Goal, Reality, Options, Will
RCT: randomised controlled trial
RESIT: REgulate your SItting Time
SPPB: Short Physical Performance Battery
T2DM: Type 2 diabetes mellitus
