# Correction: The Regulate your Sitting Time (RESIT) intervention for reducing sitting time in individuals with type 2 diabetes: findings from a randomised-controlled feasibility trial

**DOI:** 10.1186/s13098-024-01384-y

**Published:** 2024-06-28

**Authors:** Marsha L. Brierley, Angel M. Chater, Charlotte L. Edwardson, Ellen M. Castle, Emily R. Hunt, Stuart J. H. Biddle, Rupa Sisodia, Daniel P. Bailey

**Affiliations:** 1 Centre for Physical Activity in Health and Disease, College of Health, Medicine and Life Sciences, Brunel University London, Kingston Lane, Uxbridge, UB8 3PH UK; 2 Division of Sport, Health and Exercise Sciences, Department of Life Sciences, Brunel University London, Kingston Lane, Uxbridge, UB8 3PH UK; 3 Institute for Sport and Physical Activity Research, Centre for Health, Wellbeing and Behaviour Change, University of Bedfordshire, Polhill Avenue, Bedford, MK41 9EA UK; 4 Centre for Behaviour Change, University College London, 1-19 Torrington Place, London, WC1E 7HB UK; 5 Leicester Lifestyle and Health Research Group, Diabetes Research Centre, University of Leicester, Leicester General Hospital, Leicester, LE5 4PW UK; 6 NIHR Leicester Biomedical Research Centre, Leicester General Hospital, Leicester, LE5 4PW UK; 7 Physiotherapy Division, Department of Health Sciences, College of Health, Medicine and Life Sciences, Brunel University London, Kingston Lane, Uxbridge, UB8 4PH UK; 8 Curtin School of Allied Health, School of Health Sciences, Curtin University, Bentley, WA 6845 Australia; 9 Centre for Health Research, University of Southern Queensland, Springfield Central, Springfield, QLD 4300 Australia; 10 Faculty of Sport & Health Sciences, University of Jyväskylä, 40014 Jyväskylä, Finland

**Correction: Diabetology & Metabolic Syndrome (2024) 16:87** 10.1186/s13098-024-01336-6

 In Table 1 of this article [1], the data in the [Control (n = 35) Intervention (n = 35)] were calculated incorrectly. The correct version of Table 1 is given in this correction.

**Table 1:** Demographic characteristics of the participants at baseline

	Control (n = 35)	Intervention (n = 35)	All (n = 70)
Sex, n (%)			
Male	13 (37%)	18 (51%)	31 (44%)
Female	22 (63%)	17 (49%)	39 (56%)
Age (years), mean (SD)	55 (11)	60 (11)	58 (11)
Ethnicity, n (%)			
Black, Asian and minority ethnic	22 (63%)	21 (60%)	43 (61%)
White (any White background)	13 (37%)	14 (40%)	27 (39%)
Education, mean (SD)			
Secondary school (e.g., high school)	8 (23%)	10 (29%)	18 (26%)
Tertiary (e.g. university and above)	27 (77%)	25 (71%)	52 (74%)
Married/cohabiting, n (%)			
Married/living as married	27 (77%)	17 (49%)	44 (63%)
Single/separated/divorced/widowed	8 (23%)	18 (51%)	26 (37%)
Employment status, n (%)			
Disabled	0 (0%)	2 (6%)	2 (3%)
Employed full time	23 (66%)	12 (34%)	35 (50%)
Employed part time	4 (11%)	4 (11%)	8 (11%)
Retired	8 (23%)	14 (40%)	22 (31%)
Student	0 (0%)	2 (6%)	2 (3%)
Unemployed	0 (0%)	1 (3%)	1 (1%)
Had COVID prior to study start, n (%)			
No	31 (89%)	34 (97%)	65 (93%)
Yes	4 (11%)	1 (3%)	5 (7%)
Effects of the COVID-19 pandemic on work/life, n (%)			
Currently shielding	6 (17%)	11 (31%)	17 (24%)
Newly working from home	11 (31%)	8 (23%)	19 (27%)
Unemployed or retired already	6 (17%)	10 (29%)	16 (23%)
Lost their job	0 (0%)	1 (3%)	1 (1%)
Been furloughed	6 (17%)	0 (0%)	6 (9%)
Currently self-isolating	2 (6%)	2 (6%)	4 (6%)
None of the above	8 (23%)	4 (11%)	12 (17%)
Other	4 (11%)	6 (17%)	10 (14%)
Years living with type 2 diabetes^a^, mean (SD)	11 (8)	12 (11)	11 (9)

^a^Data on years living with type 2 diabetes was only available for 54 participants (n = 28 for usual care control group and n = 26 for intervention group)

The original article has been corrected.
