# Supplementary material for: The Regulate your Sitting Time (RESIT) intervention for reducing sitting time in individuals with type 2 diabetes: findings from a randomised-controlled feasibility trial
Source: Diabetol Metab Syndr. 2024 Apr 24;16:87. doi: 10.1186/s13098-024-01336-6 (PMC11040907; doi:10.1186/s13098-024-01336-6)
Supplement: Supplementary file 1 — Supplementary Material 1 [file 13098_2024_1336_MOESM1_ESM.docx]

**Supplementary Material 1.**

**Behaviour change techniques in the RESIT intervention**

| Behaviour change technique [taxonomy label] |
| --- |
| Goal setting (behaviour) [1.1] |
| Problem solving [1.2] |
| Action planning [1.4] |
| Review behaviour goals [1.5] |
| Discrepancy between current behaviour and goal [1.6] |
| Feedback on behaviour [2.2] |
| Self-monitoring of behaviour [2.3] |
| Social support – unspecified [3.1] |
| Information about antecedents [4.2] |
| Information about health consequences [5.1] |
| Demonstration of the behaviour [6.1] |
| Prompts/cues [7.1] |
| Credible source [9.1] |
| Pros and cons [9.2] |
| Social reward [10.4]  Verbal persuasion about capability [15.1] |

The behaviour change technique description and taxonomy labels are taken from the behaviour change technique taxonomy (v1) (1).

1. Michie S, Richardson M, Johnston M, Abraham C, Francis J, Hardeman W, et al. The behavior change technique taxonomy (v1) of 93 hierarchically clustered techniques: building an international consensus for the reporting of behavior change interventions. Ann Behav Med. 2013;46(1):81-95.

**Overview of self-selected behaviour change tools.**

| **Type of tool** | **Name of tool** | **Overview of tool** |
| --- | --- | --- |
| Smartphone apps | Stand up! The Work Break Timer | Apple phone app: targets reductions in prolonged sitting by encouraging regular breaks using goal setting, feedback and prompts. |
|  | Sitting Timer | Android phone app: targets reductions in prolonged sitting by encouraging regular breaks using goal setting, feedback and prompts. |
| Computer prompts | Outstanding | Google chrome extension: targets reductions in prolonged sitting by facilitating self-monitoring and encouraging regular breaks using prompts |
|  | Break Timer | Google chrome extension: targets reductions in prolonged sitting by facilitating self-monitoring and encouraging regular breaks using prompts |
|  | Workrave | Computer software: targets reductions in prolonged sitting by facilitating self-monitoring and encouraging regular breaks using prompts |
| Wearable devices | MvBii z-Track | Wrist-worn device: tracks sitting and physical activity; enables setting and monitoring of goals and provides feedback on sitting and physical activity. It targets reductions in prolonged sitting using prompts and connects to a smartphone app to access real-time feedback. |
|  | Garmin Vivofit4 | Wrist-worn device: uses prompts to reduce prolonged sitting periods, tracks sitting time, steps and calories and connects to an app to set and monitor goals and access real-time feedback. |
|  | Mi Smart band 4 | Wrist-worn device: uses prompts to reduce prolonged sitting periods, tracks sitting time, steps, heart rate, and calories and connects to an app to set and monitor goals and access real-time feedback. |
