# Supplementary material for: The Regulate your Sitting Time (RESIT) intervention for reducing sitting time in individuals with type 2 diabetes: findings from a randomised-controlled feasibility trial
Source: Diabetol Metab Syndr. 2024 Apr 24;16:87. doi: 10.1186/s13098-024-01336-6 (PMC11040907; doi:10.1186/s13098-024-01336-6)
Supplement: Supplementary file 2 — Supplementary Material 2 [file 13098_2024_1336_MOESM2_ESM.doc]

**Supplementary Material 2.** Interview schedules.

**INTERVIEW SCHEDULE (INTERVENTION)**

*The aim of this discussion is to talk about your experiences of the RESIT programme. We would like to know about your experiences of the study and how the study may or may not have changed your behaviour. The discussion will last for approximately 45-60 minutes. There are no right or wrong answers, so please speak freely and honestly as we are interested in your own opinions and experiences. We are keen to understand what works and what needs improving.*

*Would it be OK if I record the conversations, this will just mean that I won’t have to write everything down and can listen to you with my full attention. The recording will only be listened to by the research team and will be transcribed and anonymised to ensure that you cannot be identified. Also you can leave the interview at any time. Thank you.*

*I’ll just start with a few general questions and then ask you specifically about some of the components of the intervention/programme.*

***NOTE:*** *collect age and gender of participant.*

***GENERAL QUESTIONS (~20mins)***

1. What motivated you to take part in the study?

The main aim of the study was to help people with Type 2 diabetes to reduce the time they spend sitting throughout the day.

1. What impact did the RESIT programme have on your sitting behaviour throughout the day?
2. How has your sitting behaviour changed?
   1. *Prompt: At work? At home?*
3. What motivated you to make these changes?
4. Do you think you would have made any of those changes without this programme?
   1. Why might this be?
5. What worked well to support reductions in sitting time during the programme?
6. What could be improved to support reductions in sitting time?
7. Who knew you were involved in the study?
   1. *Prompt: Family? Friends? Work Colleagues?*
   2. How did they support you?
8. What should we consider for a future study that we haven’t covered?

***MEASUREMENT SESSIONS (~5mins)***

1. What do you remember about the measurements that we took as part of the study?
2. How did you feel when this data was collected?
   1. *Prompt: Did it makes sense? Anxious about tests?*
3. What should we consider when taking these measures in a future study?
4. How did having the measures taken at the beginning impact on you?
   1. *Prompt: Was it motivating in any way? What, if anything, did you change anything as a result of doing the tests?)*
   2. *Prompt: How about the follow up measurement sessions at 3 and 6 months?*

**INTERVENTION COMPONENTS**

***ONLINE EDUCATION SESSION* (~5mins)**

1. What do you remember about the online education session?
2. What are your thoughts of this session? (*prompts: What impact did the information have on you? What particular things did you find useful? Good things and bad things with the session?)*

***SELF-SELECTED TOOLS: APPS AND WEARABLE DEVICES (~10mins)***

You also had the choice of phone apps, computer apps and wearable devices to use during the study.

1. Tell me about your experiences of the phone apps, computer apps and wearable devices? *How did they influence your behaviour?*
2. How did you incorporate their use in your working day
   1. *Prompt: e.g., set goals, use at set times, memory, prompting?*
3. Can you explain if your use of these tools changed over time? Why?

***SUPPORT SESSIONS WITH HEALTH COACH (~5mins)***

You were offered support sessions with a health coach.

1. How useful did you find them?
2. What did you get out of them?
3. Suggestions for improvement?
4. If you didn’t have these sessions – is there any particular reason why?

Ending Question: Is there anything that you would like to add?

**INTERVIEW SCHEDULE (CONTROL)**

*The aim of this interview is to discuss your experiences of the project you have been involved in. We would like to get your experiences of the study and how the study may or may not have changed your behaviour. The discussion will last for approximately 20 minutes. There are no right or wrong answers, so please speak freely and honestly as we are interested in your own opinions and experiences.*

*Would it be OK if I record the conversations, this will just mean that I won’t have to write everything down and can listen to you with my full attention. The recording will only be listened to by the research team and will be transcribed and anonymised to ensure that you cannot be identified. Also you can leave the interview at any time. Thank you.*

***NOTE:*** *collect age and gender of participant.*

1. What motivated you to take part in the study?
2. Once you were told you were in the Control group of the study and were not going to receive the RESIT programme during the research study, what motivated you to stay in the study?
3. What do you remember about the measurements that we took as part of the study?
4. How did you feel when this data was collected?
   1. *Prompt: Did it makes sense? Anxious about tests?*
5. What should we consider when taking these measures in a future study?
6. How did having the measures taken at the beginning impact on you?
   1. *Prompt: Was it motivating in any way?* *What, if anything, did you change anything as a result of doing the tests?*
   2. *Prompt: How about the follow up measurement sessions at 3 and 6 months?*
7. Can you explain any changes you’ve made in your behaviours since the start of the research study? When did you make them? Why did you make them?
8. During the study did you meet anyone who was part of the RESIT intervention group?
   1. *Prompt*: *If yes, how did this influence you?*

Ending Question: Is there anything that you would like to add?

**INTERVIEW SCHEDULE (HEALTH COACHES)**

**Introduction**

*We are hoping to talk to all health coaches as we are very interested in hearing about their own experiences and views of the RESIT intervention.*

*Would it be OK if I record the conversations, this will just mean that I won’t have to write everything down and can listen to you with my full attention. The recording will only be listened to by the research team and will be transcribed and anonymised to ensure that you cannot be identified. Also, you can leave the interview at any time. Thank you.*

*The aim is to provide themes around issues of interest related to the health coaching aspect of the RESIT intervention and any other issues of importance to health coaches.*

**The Guide:**

Opening questions

How many clients have you had sessions with in the RESIT programme?

The RESIT Programme

- What are your impressions of the RESIT programme?
- What do you think the participants thought about the RESIT programme?
- How successful were clients in making changes on the RESIT programme?
  - How do you feel the RESIT programme changed their behaviours?

Delivery

- What aspects of the sessions did you find most easy?
- What aspects of the sessions did you find most challenging?
- What recommendations do you have for improving the delivery of the sessions?

Training

- Overall, what was your training experience like?
- What things can you remember from the training session?
  - *Prompt: Which techniques do you remember?*
- What aspects of the training did you find most useful?
- What aspects of the training did you find most challenging?
- How did the training change your practice as a health coach?
  - *Prompt: Delivery style? Questions/approach used?*
- Which specific techniques have you used in your practice since you attended the training?
  - *Prompt: What examples can you give me where this worked well?*
  - *Prompt: What didn’t work so well?*
- How would you judge your confidence with using Motivational Interviewing skills after attending the training session?
- What else would you like to learn around this topic area?
- How would you rate your overall satisfaction with the training session that you attended?

Ending Question: Is there anything that you would like to add?
