# Supplementary material for: The Regulate your Sitting Time (RESIT) intervention for reducing sitting time in individuals with type 2 diabetes: findings from a randomised-controlled feasibility trial
Source: Diabetol Metab Syndr. 2024 Apr 24;16:87. doi: 10.1186/s13098-024-01336-6 (PMC11040907; doi:10.1186/s13098-024-01336-6)
Supplement: Supplementary file 3 — Supplementary Material 3 [file 13098_2024_1336_MOESM3_ESM.docx]

**Supplementary Material 3.**

**Self-reported effects of study measurements on behaviour in the intervention group.**

|  | at 3 months | | at 6 months | |
| --- | --- | --- | --- | --- |
|  | N | %* | N | %‡ |
| The offer of study measurements encouraged me to participate in the study |  |  |  |  |
| Strongly agree | 4 | 18% | 4 | 18% |
| Agree | 11 | 50% | 9 | 41% |
| Neither agree nor disagree | 5 | 23% | 7 | 32% |
| Disagree | 2 | 9% | 0 | 0% |
| Strongly disagree | 0 | 0% | 2 | 9% |
|  |  |  |  |  |
| The measurements at the start of the study motivated me to change aspects of my behaviour |  |  |  |  |
| Strongly agree | 2 | 9% | 3 | 14% |
| Agree | 15 | 68% | 13 | 59% |
| Neither agree nor disagree | 4 | 18% | 4 | 18% |
| Disagree | 1 | 5% | 0 | 0% |
| Strongly disagree | 0 | 0% | 2 | 9% |
|  |  |  |  |  |
| The measurements at the start of the study motivated me to want to change how much time I spent sitting |  |  |  |  |
| Strongly agree | 7 | 32% | 4 | 18% |
| Agree | 11 | 50% | 14 | 64% |
| Neither agree nor disagree | 3 | 14% | 2 | 9% |
| Disagree | 1 | 5% | 0 | 0% |
| Strongly disagree | 0 | 0% | 2 | 9% |
|  |  |  |  |  |
| Knowing that follow up measurements would be taken later in the study motivated me to want to change aspects of my behaviour |  |  |  |  |
| Strongly agree | 5 | 23% | 3 | 14% |
| Agree | 10 | 45% | 15 | 68% |
| Neither agree nor disagree | 5 | 23% | 2 | 9% |
| Disagree | 2 | 9% | 0 | 0% |
| Strongly disagree | 0 | 0% | 2 | 9% |
|  |  |  |  |  |
| Knowing that follow up measurements would be taken later in the study motivated me to want to change how much time I spent sitting |  |  |  |  |
| Strongly agree | 6 | 27% | 3 | 14% |
| Agree | 10 | 45% | 14 | 64% |
| Neither agree nor disagree | 4 | 18% | 3 | 14% |
| Disagree | 2 | 9% | 0 | 0% |
| Strongly disagree | 0 | 0% | 2 | 9% |

* % is calculated as number of responses divided by number of participants that fully completed the 3-month survey (N=22) multiplied by 100.

‡ % is calculated as number of responses divided by number of participants that fully completed the 6-month survey (N=22) multiplied by 100.

**Self-reported effects of study measurements on behaviour in the control group.**

|  | 3 months | | 6 months | |
| --- | --- | --- | --- | --- |
|  | N | %\|\| | N | %# |
| The offer of study measurements encouraged me to participate in the study |  |  |  |  |
| Strongly agree | 0 | 0% | 2 | 7% |
| Agree | 10 | 48% | 10 | 34% |
| Neither agree nor disagree | 11 | 52% | 14 | 48% |
| Disagree | 0 | 0% | 3 | 10% |
| Strongly disagree | 0 | 0% | 0 | 0% |
|  |  |  |  |  |
| The measurements at the start of the study motivated me to change aspects of my behaviour |  |  |  |  |
| Strongly agree | 0 | 0% | 2 | 7% |
| Agree | 6 | 29% | 10 | 34% |
| Neither agree nor disagree | 12 | 57% | 11 | 38% |
| Disagree | 3 | 14% | 5 | 17% |
| Strongly disagree | 0 | 0% | 1 | 3% |
|  |  |  |  |  |
| The measurements at the start of the study motivated me to want to change how much time I spent sitting |  |  |  |  |
| Strongly agree | 0 | 0% | 2 | 7% |
| Agree | 9 | 43% | 12 | 41% |
| Neither agree nor disagree | 10 | 48% | 11 | 38% |
| Disagree | 2 | 10% | 4 | 14% |
| Strongly disagree | 0 | 0% | 0 | 0% |
|  |  |  |  |  |
| Knowing that follow up measurements would be taken later in the study motivated me to want to change aspects of my behaviour |  |  |  |  |
| Strongly agree | 0 | 0% | 1 | 3% |
| Agree | 8 | 38% | 12 | 41% |
| Neither agree nor disagree | 12 | 57% | 9 | 31% |
| Disagree | 1 | 5% | 6 | 21% |
| Strongly disagree | 0 | 0% | 1 | 3% |
|  |  |  |  |  |
| Knowing that follow up measurements would be taken later in the study motivated me to want to change how much time I spent sitting |  |  |  |  |
| Strongly agree | 0 | 0% | 1 | 3% |
| Agree | 7 | 33% | 11 | 38% |
| Neither agree nor disagree | 12 | 57% | 11 | 38% |
| Disagree | 2 | 10% | 5 | 17% |
| Strongly disagree | 0 | 0% | 1 | 3% |

|| % is calculated as number of responses divided by number of participants that fully completed the 3-month survey (N=21) multiplied by 100.

# % is calculated as number of responses divided by number of participants that fully completed the 6-month survey (N=29) multiplied by 100.
