# Supplementary material for: The Regulate your Sitting Time (RESIT) intervention for reducing sitting time in individuals with type 2 diabetes: findings from a randomised-controlled feasibility trial
Source: Diabetol Metab Syndr. 2024 Apr 24;16:87. doi: 10.1186/s13098-024-01336-6 (PMC11040907; doi:10.1186/s13098-024-01336-6)
Supplement: Supplementary file 4 — Supplementary Material 4 [file 13098_2024_1336_MOESM4_ESM.docx]

| **Supplementary Material 4.** Nested qualitative sample characteristics (n=30) | | | | | | | |
| --- | --- | --- | --- | --- | --- | --- | --- |
|  |  |  |  |  |  |  |  |
| **Study ID** | **Group** | **Age** | **Sex** | **Ethnicity** | **Education** | **Relationship status** | **Employment status** |
| RS02 | Control | 64 | F | White | Tertiary | Married | Employed full-time |
| RS55 | Control | 61 | F | Asian | Tertiary | Widowed | Retired |
| RS32 | Control | 72 | M | White | Tertiary | Married | Employed full-time |
| RS09 | Control | 57 | F | White | Tertiary | Married | Retired |
| RS25 | Control | 37 | M | Other | Tertiary | Single | Employed full-time |
| RS65 | Control | 60 | F | White | Tertiary | Married | Retired |
| RS23 | Control | 61 | M | White | Tertiary | Married | Retired |
| RS46 | Control | 43 | M | White | Tertiary | Living as married | Employed full-time |
| RS60 | Control | 53 | M | White | Tertiary | Single | Employed full-time |
| RS64 | Control | 50 | F | White | Tertiary | Living as married | Employed part-time |
| RS70 | Control | 71 | M | White | Tertiary | Married | Employed full-time |
| RS30 | Control | 58 | F | White | Secondary school | Married | Employed full-time |
| RS13 | Intervention | 65 | M | White | Tertiary | Married | Employed full-time |
| RS22 | Intervention | 62 | M | Asian | Tertiary | Married | Employed full-time |
| RS31 | Intervention | 82 | M | Asian | Tertiary | Married | Retired |
| RS14 | Intervention | 47 | F | Asian | Tertiary | Married | Employed full-time |
| RS42 | Intervention | 63 | M | White | Tertiary | Single | Retired |
| RS44 | Intervention | 84 | F | White | Secondary school | Widowed | Retired |
| RS49 | Intervention | 67 | M | White | Tertiary | Widowed | Retired |
| RS07 | Intervention | 49 | F | White | Tertiary | Single | Student |
| RS63 | Intervention | 71 | F | Mixed | Tertiary | Divorced | Retired |
| RS11 | Intervention | 67 | F | White | Tertiary | Widowed | Retired |
| RS47 | Intervention | 52 | F | Asian | Tertiary | Married | Employed part-time |
| RS68 | Intervention | 63 | F | Asian | Secondary school | Married | Disabled |
| RS04 | Intervention | 53 | M | White | Tertiary | Divorced | Retired |
| HC1 | Health coach | 27 | F | - | - | - | - |
| HC2 | Health coach | 32 | F | - | - | - | - |
| HC3 | Health coach | - | F | - | - | - | - |
| HC4 | Health coach | 26 | F | - | - | - | - |
| HC5 | Health coach | 32 | M | - | - | - | - |
| N.B. Other than age and sex, no other demographic characteristics were collected for the health coaches | | | | | | | |
|  |  |  |  |  |  |  |  |
|  |  |  |  |  |  |  |  |
