# Supplementary material for: The Regulate your Sitting Time (RESIT) intervention for reducing sitting time in individuals with type 2 diabetes: findings from a randomised-controlled feasibility trial
Source: Diabetol Metab Syndr. 2024 Apr 24;16:87. doi: 10.1186/s13098-024-01336-6 (PMC11040907; doi:10.1186/s13098-024-01336-6)
Supplement: Supplementary file 5 — Supplementary Material 5 [file 13098_2024_1336_MOESM5_ESM.docx]

**Supplementary Material 5.** Health and wellbeing outcomes.

**Descriptive statistics for waist circumference and physical function**

|  |  | **Baseline** | | **3 months** | | **6 months** | | **Change (baseline to 3 months)** | | **Change (baseline to 6 months)** | |
| --- | --- | --- | --- | --- | --- | --- | --- | --- | --- | --- | --- |
|  |  | **Control** | **Intervention** | **Control** | **Intervention** | **Control** | **Intervention** | **Control** | **Intervention** | **Control** | **Intervention** |
| **Variable** |  | **(n = 32)** | **(n = 30)** | **(n = 32)** | **(n = 30)** | **(n = 32)** | **(n = 30)** | **(n = 32)** | **(n = 30)** | **(n = 32)** | **(n = 30)** |
| Waist circumference (cm) | Mean | 104.9 | 104.7 | 104.9 | 102.4 | 103.3 | 100.8 | 0.1 | -2.3 | -1.5 | -3.9 |
|  | SD | 13.6 | 13.5 | 14.5 | 12.5 | 13.2 | 13.5 | 5.0 | 13.8 | 5.4 | 13.5 |
| Sit-to-stand score | Mean | 3.9 | 3.7 | 4.0 | 3.6 | 3.5 | 3.4 | 0.1 | 0.0 | -0.4 | -0.2 |
|  | SD | 0.4 | 1.0 | 0.0 | 0.9 | 0.6 | 1.0 | 0.4 | 0.8 | 0.8 | 1.0 |
| Balance score | Mean | 4.0 | 3.8 | 4.0 | 3.8 | 4.0 | 3.8 | -0.1 | 0.0 | 0.0 | 0.0 |
|  | SD | 0.0 | 0.8 | 0.0 | 0.8 | 0.0 | 0.9 | 0.7 | 0.0 | 0.0 | 0.2 |
| Walk score | Mean | 3.6 | 3.5 | 3.7 | 3.4 | 3.8 | 3.4 | 0.1 | -0.2 | 0.3 | -0.1 |
|  | SD | 0.6 | 0.8 | 0.6 | 0.9 | 0.4 | 0.9 | 0.9 | 1.3 | 0.8 | 1.2 |
| SPPB score | Mean | 11.5 | 11.0 | 11.6 | 10.8 | 11.4 | 10.6 | 0.1 | -0.2 | -0.1 | -0.4 |
|  | SD | 0.7 | 1.6 | 1.0 | 2.4 | 0.8 | 2.3 | 1.3 | 1.7 | 1.2 | 1.8 |

SPPB, Short Physical Performance Battery. Data are presented for participants who completed the questionnaires at all three timepoints.

**Descriptive statistics for health, wellbeing and psychological questionnaire measures**

|  |  |  | **Baseline** | | **3-month follow-up** | | **6-month follow-up** | | **Change (baseline to 3 months)** | | **Change (baseline to 6 months)** | |
| --- | --- | --- | --- | --- | --- | --- | --- | --- | --- | --- | --- | --- |
|  |  |  | **Control** | **Intervention** | **Control** | **Intervention** | **Control** | **Intervention** | **Control** | **Intervention** | **Control** | **Intervention** |
| **Variable** |  |  | **(n = 24)** | **(n = 25)** | **(n = 24)** | **(n = 25)** | **(n = 24)** | **(n = 25)** | **(n = 24)** | **(n = 25)** | **(n = 24)** | **(n = 25)** |
| WHOQOL-BREF | |  |  |  |  |  |  |  |  |  |  |  |
| Physical (%) | | Mean | 15.7 | 14.7 | 15.5 | 15.3 | 15.9 | 15.4 | -0.2 | 0.6 | 0.2 | 0.7 |
|  |  | SD | 2.6 | 3.2 | 2.6 | 3.4 | 2.4 | 3.5 | 1.6 | 1.9 | 1.9 | 1.5 |
| Psychological (%) | | Mean | 14.8 | 13.2 | 14.4 | 14.2 | 15.0 | 14.4 | -0.4 | 1.0 | 0.2 | 1.2 |
|  |  | SD | 2.5 | 3.0 | 2.2 | 2.8 | 2.3 | 3.2 | 1.8 | 2.2 | 1.8 | 2.5 |
| Social relationships (%) | | Mean | 15.0 | 13.6 | 13.9 | 14.0 | 14.7 | 14.1 | -1.1 | 0.4 | -0.3 | 0.5 |
|  |  | SD | 3.5 | 2.6 | 3.8 | 3.6 | 3.3 | 3.7 | 2.0 | 2.5 | 2.1 | 3.0 |
| Environment (%) | | Mean | 15.9 | 14.6 | 16.0 | 15.6 | 16.2 | 15.9 | 0.1 | 1.0 | 0.3 | 1.3 |
|  |  | SD | 2.1 | 2.9 | 2.3 | 2.5 | 2.1 | 3.1 | 1.5 | 2.1 | 1.2 | 2.5 |
| Sitting self-efficacy | | Mean | 26.5 | 26.4 | 26.8 | 29.2 | 28.5 | 29.0 | 0.3 | 2.8 | 2.0 | 2.6 |
|  |  | SD | 7.3 | 6.5 | 5.5 | 5.1 | 6.9 | 4.5 | 6.8 | 9.8 | 8.2 | 9.0 |
| General self-efficacy | | Mean | 33.3 | 31.8 | 31.9 | 32.1 | 32.2 | 31.9 | -1.4 | 0.4 | -1.1 | 0.1 |
|  |  | SD | 4.2 | 3.5 | 5.0 | 4.3 | 4.7 | 3.9 | 4.7 | 3.5 | 4.5 | 3.4 |
| Perceived stress |  | Mean | 13.3 | 16.0 | 14.8 | 15.0 | 13.3 | 15.2 | 1.6 | -1.0 | 0.0 | -0.8 |
|  |  | SD | 5.8 | 6.3 | 6.6 | 6.1 | 7.9 | 6.5 | 5.6 | 4.6 | 6.7 | 4.9 |
| WHO-5 wellbeing (%) | | Mean | 63.8 | 51.7 | 58.0 | 57.0 | 64.7 | 62.1 | -5.8 | 5.3 | 0.8 | 10.4 |
|  |  | SD | 12.8 | 18.0 | 13.9 | 22.4 | 17.5 | 23.2 | 13.1 | 17.6 | 12.4 | 22.2 |
| Negative affect |  | Mean | 18.7 | 23.0 | 16.6 | 17.3 | 16.0 | 17.8 | -2.1 | -5.7 | -2.7 | -5.2 |
|  |  | SD | 8.6 | 11.9 | 6.1 | 7.0 | 5.9 | 6.3 | 9.4 | 11.5 | 9.9 | 11.5 |
| Positive affect |  | Mean | 34.3 | 33.2 | 32.6 | 33.2 | 36.6 | 34.1 | -1.7 | 0.0 | 2.3 | 0.9 |
|  |  | SD | 6.4 | 7.2 | 6.1 | 7.7 | 6.6 | 8.3 | 5.7 | 5.9 | 5.2 | 8.2 |
| Fatigue |  | Mean | 10.0 | 11.8 | 11.2 | 9.7 | 8.9 | 11.0 | 1.1 | -2.1 | -1.2 | -0.8 |
|  |  | SD | 4.4 | 4.4 | 4.5 | 3.1 | 3.5 | 6.0 | 5.6 | 4.9 | 4.5 | 4.7 |
| Musculoskeletal trouble | |  | (n=23) | (n=24) | (n=23) | (n=24) | (n=23) | (n=24) | (n=23) | (n=24) | (n=23) | (n=24) |
| During last 12 months | | n | 20 | 20 | 20 | 18 | 21 | 19 | - | - | - | - |
|  |  | % | 87% | 83% | 87% | 75% | 91% | 79% | 0% | -8% | 4% | -4% |
| During last 7 days | | n | 13 | 11 | 16 | 15 | 16 | 13 | - | - | - | - |
|  |  | %* | 65% | 55% | 80% | 83% | 76% | 68% | 15% | 28% | 11% | 13% |

WHOQOL-BREF, World Health Organization Quality of Life brief version; WHO-5: World Health Organisation-five wellbeing index. Data are presented for participants who completed the questionnaires at all three timepoints.

WHOQOL-BREF – comprised of four derived domain scores that produce a raw quality of life profile. The raw score is multiplied by four to produce a percentage (0-100%). A higher score indicates higher quality of life.

Sitting self-efficacy – ten items are rated on a 4-point Likert scale from 1 (very uncertain) to 4 (very certain) and summed to produce an overall score (range 10-40). A higher score indicates higher perceived sitting self-efficacy.

General self-efficacy – ten items are rated on a 4-point Likert scale from 1 (not at all true) to 4 (exactly true) and summed to produce an overall score (range 10-40). A higher score indicates higher perceived general self-efficacy.

Perceived stress – ten items are rated on a 5-point Likert scale from 0 (never) to 4 (almost always). Positively worded items are reverse scored and ratings are then summed to produce an overall score (range 0-40). A higher score indicates higher perceived stress.

WHO-5 wellbeing – five items are rated on a 6-point Likert scale from 0 (at no time) to 5 (all of the time) and summed to produce a raw score (range 0-25). The raw score is multiplied by four to produce a percentage (0-100%). A higher score indicates increased wellbeing.

Negative and positive affect scale – twenty items are rated on a 5-point Likert scale from 1 (Very slightly or not at all) to 5 (extremely). Scores are summed separately for the 10 positive and 10 negative items (range for each scale 10-50). Lower scores indicate low (positive or negative) affect and higher scores indicate high affect.

Fatigue – ten items are rated on a 4-point Likert scale from 0 (less than usual) to 3 (much more than usual) and one item rated 0 (better than usual) to 3 (much worse than usual). Ratings are summed to produce an overall score (range 0-33). A higher score indicates worse perceived fatigue.

**Descriptive statistics for Pittsburgh Sleep Quality Index**

|  |  | **Baseline** | | **3 months** | | **6 months** | | **Change (baseline to 3 months)** | | **Change (baseline to 6 months)** | |
| --- | --- | --- | --- | --- | --- | --- | --- | --- | --- | --- | --- |
|  |  | Control | Intervention | Control | Intervention | Control | Intervention | Control | Intervention | Control | Intervention |
| **Variable** |  | (n = 23) | (n = 23) | (n = 23) | (n = 23) | (n = 23) | (n = 23) | (n = 23) | (n = 23) | (n = 23) | (n = 23) |
| Duration of sleep | Mean | 1.0 | 0.7 | 1.0 | 0.7 | 0.7 | 0.7 | 0.0 | 0.0 | -0.3 | 0.0 |
|  | SD | 1.1 | 1.0 | 1.0 | 1.0 | 0.6 | 1.1 | 0.5 | 0.7 | 0.7 | 0.9 |
| Sleep disturbances | Mean | 1.0 | 1.3 | 1.1 | 1.3 | 1.1 | 1.1 | 0.0 | 0.0 | 0.1 | -0.2 |
|  | SD | 0.6 | 0.4 | 0.4 | 0.5 | 0.3 | 0.5 | 0.5 | 0.6 | 0.6 | 0.6 |
| Sleep latency | Mean | 1.3 | 1.5 | 1.3 | 1.2 | 1.0 | 0.8 | 0.0 | -0.3 | -0.2 | -0.7 |
|  | SD | 0.9 | 1.0 | 1.0 | 0.9 | 1.0 | 0.7 | 0.7 | 1.0 | 0.7 | 1.0 |
| Daytime dysfunction | Mean | 0.7 | 0.8 | 0.9 | 0.7 | 0.6 | 0.8 | 0.2 | -0.1 | -0.1 | 0.0 |
|  | SD | 0.7 | 0.7 | 0.6 | 0.6 | 0.6 | 0.7 | 0.6 | 0.6 | 0.7 | 0.8 |
| Habitual sleep efficiency | Mean | 0.7 | 1.3 | 0.7 | 1.1 | 0.6 | 1.0 | 0.0 | -0.1 | -0.2 | -0.2 |
|  | SD | 1.1 | 1.3 | 1.1 | 1.2 | 0.8 | 1.2 | 0.9 | 1.3 | 0.8 | 1.4 |
| Subjective sleep quality | Mean | 1.2 | 1.0 | 1.1 | 1.0 | 1.1 | 1.0 | -0.1 | -0.1 | -0.1 | -0.1 |
|  | SD | 0.9 | 0.8 | 0.8 | 0.7 | 0.8 | 0.8 | 0.7 | 0.6 | 0.7 | 0.5 |
| Need medication to sleep | Mean | 0.4 | 0.5 | 0.6 | 0.5 | 0.3 | 0.7 | 0.1 | 0.0 | -0.1 | 0.2 |
|  | SD | 1.0 | 1.2 | 1.2 | 1.2 | 0.9 | 1.3 | 1.1 | 0.9 | 0.7 | 1.1 |
| PSQI global score | Mean | 6.3 | 7.1 | 6.6 | 6.4 | 5.4 | 6.0 | 0.3 | -0.7 | -0.9 | -1.0 |
|  | SD | 3.9 | 3.9 | 4.4 | 4.3 | 3.0 | 3.7 | 2.1 | 2.9 | 2.2 | 3.5 |

Data are presented for participants who completed the questionnaire at all three timepoints. The Pittsburgh Sleep Quality Index measures self-rated sleep quality experienced in the last month with a higher score indicating worse sleep quality. There are seven derived components (scored from 0–3, no difficulty to severe difficulty) and summation of component scores produces a global score (range 0 to 21).
